# Arthroscopic versus open treatment for femoroacetabular impingement: A systematic review and meta-analyses

**DOI:** 10.1097/MD.0000000000023364

**Published:** 2020-11-20

**Authors:** Hu-Yun Qiao, Yong-Hong Zhang, Yi-Ming Ren, Meng-Qiang Tian

**Affiliations:** a Department of Orthopedics, The Second Hospital of Shanxi Medical University, Taiyuan; b Department of Joint and Sport Medicine, Tianjin Union Medical Center, Tianjin, PR China.

**Keywords:** femoroacetabular impingement, hip-arthroscopy, meta-analysis, surgical hip dislocation, systematic review

## Abstract

**Background:**

Femoroacetabular impingement (FAI) is a common cause of hip pain and restricted range of motion in young adults and athletes. This study aims to compare clinical results and complications between patients treated for FAI who underwent either arthroscopic or open treatment.

**Methods:**

The 7 studies were acquired from PubMed, Medline, Embase, and Cochrane Library. The data were extracted analyzed by RevMan5.3. Mean differences (MDs), odds ratios (ORs), and 95% confidence intervals (CIs) were calculated. The Newcastle–Ottawa Scale were used to assess risk of bias.

**Results:**

Seven observational studies were assessed. The methodological quality of the trials indicated a low risk of bias. The pooled results of the modified Harris Hip Score (mHHS), the Non-Arthritic Hip Score (NAHS), the Visual Analogue Scale (VAS), and satisfaction rate showed that the differences were not statistically significant between arthroscopic treatment (AT) and open treatment (OT). The difference of postoperative alpha angle was statistically significant, and OT was more effective [MD = 3.08, 95% confidence interval (95% CI) = 1.45–4.70, *P* = .0002]. The difference of postoperative internal rotation angle was statistically significant, and OT had better internal rotation angle (MD = -3.21, 95% CI = -6.14 to -0.28, *P* = .03). However, the difference of complications was statistically significant and AT achieved better result than OT (OR = 0.41, 95% CI = 0.22–0.74, *P* =0.003).

**Conclusion:**

AT had comparable effect and lower complications than OT, but had less improvement in alpha angle and internal rotation angle.

## 1 Introduction

Femoroacetabular impingement (FAI) is a common cause of hip pain and restricted range of motion in young adults and athletes, which is defined as the abnormal contact between the acetabular rim and femoral neck, involving either the femur (cam), the acetabulum (pincer), or both types.^[1–4]^ The repeated abnormal contact would further contribute to chondrolabral lesion and early hip osteoarthritis.^[5]^ Conservative treatment can be attempted initially, which consists of modifying high-impact physical activities, avoiding weighted exercises associated with excessive flexion and torsion movements, and, finally, the use of anti-inflammatory medications. Surgical treatment is indicated in cases when conservative treatment brings only temporary relief.^[6–9]^

Open surgical hip dislocation has long been the standard surgical modality for treating FAI.^[10]^ However, technical advances have enabled an arthroscopic approach for treatment of FAI. In the last few years, we have seen a substantial increase in the number of published studies showing promising midterm results comparing the arthroscopic treatment (AT) and open treatment (OT) of FAI. Especially, in a meta-analysis, Zhang et al^[11]^ evaluated the efficacy and safety of hip arthroscopy versus open surgical dislocation for treating FAI through 5 published clinical trials, revealing that hip arthroscopy resulted in higher Non-Arthritic Hip Score (NAHS) and lower reoperation rates, but had less improvement in alpha angle in patients with cam osteoplasty, than open surgical dislocation. In this updated article, we included 7 relevant studies to compare the clinical outcomes and complications of AT versus OT in FAI to provide more believable evidence for clinical decision making.

## 2 Materials and methods

Ethical approval or patient consent was not required, as the present study was a review of previous published literatures.

### 2.1 Inclusive criteria of published studies

#### 2.1.1 Types of studies

We considered all published and unpublished studies covering randomized controlled trials (RCTs), and observational studies, including retrospective and prospective studies. English is a common language and most databases are in English. So, only English language studies were considered.

#### 2.1.2 Types of participants

All patients had been diagnosed as FAI, regardless of the diagnostic criteria used, etiology of the disease, associated pathology, gender, and age.

#### 2.1.3 Types of interventions

All surgical techniques, including the hip arthroscopy technique and open treatment or surgical hip dislocation, were considered. The exclusion criteria were as follows: insufficient clinical outcome data in studies and reviews, letters, or conference articles.

#### 2.1.4 Types of outcome measures

The primary outcome measures were the clinical outcomes synthesizing the modified Harris Hip Score (mHHS), the NAHS, the Visual Analogue Scale (VAS), and satisfaction rate. The secondary outcomes included postoperative alpha angle, postoperative internal rotation angle, and complications.

#### 2.1.5 Search methods for identification of studies

Four databases (PubMed, Medline, Embase, and Cochrane Library) were searched using the terminology: (“femoracetabular” [MeSH term] OR “femoro-acetabular” OR “femoro acetabular”) AND (impingement [MeSH term] OR “impingement syndrome”) AND (surgery [MeSH term] OR surgeries OR surgical OR arthroscopy OR arthroscopies OR arthroscopic OR operative OR osteotomy OR osteotomies OR dislocation OR procedure) through April 2018 to collect relevant studies about the clinical comparisons of AT versus OT in FAI. The other sources (such as Google Scholar and Controlled Trials metaRegister) were searched using the keywords femoroacetabular impingement, surgery, treatment, therapy, complications, adverse effect, randomized controlled trial, and clinical trial. The titles and abstracts of potential related articles identified by the electronic search were reviewed. References from retrieved articles were also assessed to extend the search strategy.

#### 2.1.6 Data collection and quality assessment

Two partners (HYQ, YMR) independently assessed the titles and abstracts of all the studies screened during initial search, and they excluded any clearly irrelevant studies using the inclusion criteria. Data were independently extracted using a standard data form for the first author's name, year of publication, sample size, gender, age, intervention, type, country, study design, follow-up, and relevant outcomes. A third partner (MQT) would handle any disagreement about inclusion of a study and reach a consensus. Observational studies were assessed by the Newcastle--Ottawa Scale including 8 items. A higher overall score indicates a lower risk of bias and a score of 5 or less (out of 9) corresponds to a high risk of bias.

### 2.2 Statistical analysis

RevMan statistical software5.3 was used for meta-analysis. The continuous variables would be conducted by mean difference (MD) and 95% confidence interval (95% CI). For the dichotomous outcome, we calculated the odds ratios (ORs) and 95% CIs. The Chi-squared statistic and the *I*^2^ statistic were used for the test of heterogeneity. A *P* < .05, *I*^2^ > 50% was considered a significant heterogeneity, and random-effect models were applied. Otherwise, fixed-effect models were used if there was no significant heterogeneity (*P* ≥ .05, *I*^2^ ≤ 50%).^[12]^ We also performed sensitivity analysis by omitting 1 study at a time to test the stability of the pooled results. Publication bias was showed by the funnel plot.

## 3 Results

### 3.1 Studies identification and inclusion

**Figure 1:**
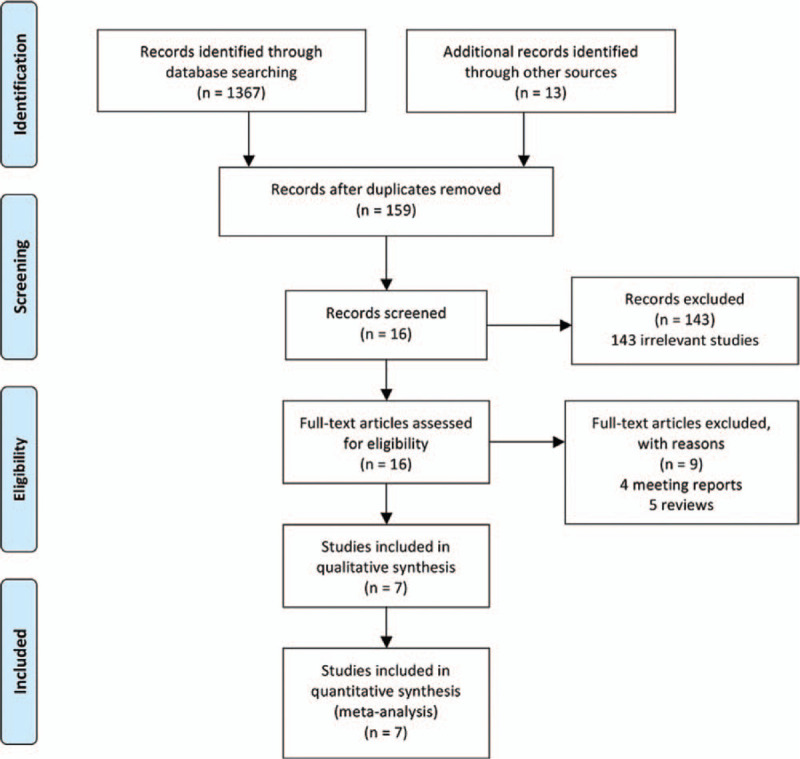
PRISMA flow diagram.

Searches conducted in the PubMed, Medline, Embase, Cochrane Library databases, and other sources, yielded a total of 1380 articles. After removing duplicates, 159 literatures remained. On the basis of the titles and abstracts review, 143 irrelevant articles of them were excluded. Sixteen full-text articles were assessed for eligibility. However, 9 articles were excluded based on the previously established exclusion criteria (4 meeting reports and 5 reviews). Finally, 7 observational studies were included in this systematic review and meta-analysis. The detail of selection process is listed in Figure 1.

### 3.2 Study characteristics

We assessed 7 studies^[13–19]^ including 3 retrospective studies and 4 prospective cohort studies in this article. The included studies were conducted in 4 countries (Switzerland, Portugal, Brazil and USA) from 2011 to 2018, and involved 606 patients (299 patients treated with AT, 307 patients treated with OT) aged 19 to 36.12 years. The average follow-up duration ranged from 12 to 59 months. The clinical outcomes of the studies were evaluated mainly based on the pooled results of mHHS, NAHS, VAS, satisfaction rate, postoperative alpha angle, postoperative internal rotation angle, and complications. The detailed information of included studies is summarized in Table 1.

**Table 1:** Characteristics of studies included.

					Intervention						
	Year	Sample size (A/O)	Female (%)	Mean age, yr	A	O	Type	Country	Study design	Follow-up, mo	Relevant outcome
Rego et al^[13]^	2018	102/96	44%	33 (18–49)	hip	surgical hip dislocation	198 cam	Portugal	Retrospective	59 (24–132)	alpha angle; NAHS; complications
					arthroscopy				Study		
Roos et al^[14]^	2017	40/16	A87%	A36.12 ± 8.7	arthroscopy	open surgery	41 cam	Brazil	retrospective case–control study	A29.1 (24–42)	alpha angle; NAHS; NHI; CEθ
			O68.75%	O35.76 ± 9.5			17 mixed			O52 (43–74)	mHHS; good or excellent;
											internal rotation; complications
Boster et al^[15]^	2014	18/5	100%	A20.1	arthroscopic treatment	open surgical dislocation	UR	USA	prospective	14.7 (12–25)	NAHS; HOS-ADL; HOS-SSS;
				O18.1					study		mHHS; complications
Domb et al^[16[^	2013	20/10	20%	A19.6	arthroscopic treatment	surgical dislocation of the hip	29 pincer	USA	prospective	A25.5 (21–34)	alpha angle; NAHS; HOS-ADL;
				O19			21 cam		matched-pair study	O24.8 (12–39)	HOS-SSS; VAS; satisfaction; mHHS;
							20 mixed				good or excellent; complications
Zingg et al^[17]^	2013	23/15	A21.7%	A27.6 ± 8.4	hip arthroscopy	surgical	7 pincer	Switzerland	prospective comparative study	12	alpha angle; mHHS; VAS;
			O26.7%	O28.9 ± 8.0		hip dislocation	2 cam				internal rotation; complications
							29 mixed				
Buchler et al^[18]^	2013	66/135	A74.2%	A33.8 (11.9–62.7)	hip arthroscopy	surgical dislocation	Cam or mixed	Switzerland	retrospective	16.7 (2–79)	alpha angle; gamma angle;
			O67.4%	O31.2 (16–54)					study		complications
Bedi et al^[19]^	2011	30/30	UR	UR	hip arthroscopy	surgical	UR	USA	prospective	UR	alpha angle; gamma angle
						hip dislocation			matched-pair study		

A = arthroscopy, CEθ = center-edge angle, HOA-SSS = the Hip Outcome Score-Sport-Specific Subscale, HOS-ADL = the Hip Outcome Score-Activities of Daily Living, mHHS = the modified Harris Hip Score, NAHS = the Non-Arthritic Hip Score, NHI = lateral view head-neck index, O = open surgery, UR = un-reported, VAS = Visual Analogue Scale.

### 3.3 Methodological assessment of study quality

Methodological quality assessment of the 7 included studies is presented in Table 2. Among the observational studies, the Newcastle–Ottawa Scale, including the exposed cohort, the nonexposed cohort, ascertainment of exposure, outcome of interest, comparability, assessment of outcome, length of follow-up, and adequacy of follow-up, was used to assess the risk of bias. The scores of all 7 studies ranged from 7 to 9, indicating a low risk of bias.

**Table 2:** Risk of bias was assessed using the Newcastle-Ottawa Scale.

		Selection					Outcome		
	Exposed	Noexposed	Ascertainment	Outcome		Assessment	Length of	Adequacy of	Total
Study	Cohort	Cohort	of exposure	of interest	Comparability	of outcome	follow-Up	follow-Up	score
Rego et al^[13]^	^∗^	^∗^	^∗^	^∗^	^∗^	^∗^	^∗^	^∗^	8
Roos et al^[14]^	^∗^	^∗^	^∗^	^∗^	^∗^	^∗^	^∗^	^∗^	8
Boster et al^[15]^	^∗^	^∗^	^∗^	^∗^	^∗^	^∗^	^∗^	–	7
Domb et al^[16]^	^∗^	^∗^	^∗^	^∗^	^∗∗^	^∗^	^∗^	^∗^	9
Zingg et al^[17]^	^∗^	^∗^	^∗^	^∗^	^∗∗^	^∗^	^∗^	–	8
Buchler et al^[18]^	^∗^	^∗^	^∗^	^∗^	^∗^	^∗^	^∗^	^∗^	8
Bedi et al^[19]^	^∗^	^∗^	^∗^	^∗^	^∗∗^	^∗^	^∗^	–	8

∗ Risk of bias was assessed using the Newcastle--Ottawa Scale. A higher overall score indicates a lower risk of bias; a score of 5 or less (out of 9) corresponds to a high risk of bias.

### 3.4 Comparison of mHHS between AT and OT

**Figure 2:**
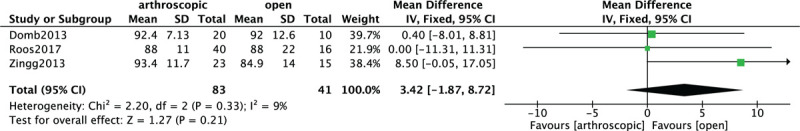
Forest plot of comparison: the modified Harris Hip Score (mHHS) between arthroscopic treatment (AT) and open treatment (OT).

Comparison of postoperative mHHS between AT and OT was conducted among the 3 included studies,^[14,16,17]^ which included 124 patients (83 patients receiving AT and 41 patients receiving OT), as shown in Figure 2. Heterogeneity testing showed that there was low heterogeneity among the studies (*P* = .33, *I*^2^ = 9%), so the fixed-effect model was used to pool the data from the 3 studies. The pooled result showed that the difference was not statistically significant between the AT group and the OT group (MD = 3.42,95% CI = -1.87 to 8.72,*P* = .21).

### 3.5 Comparison of NAHS between AT and OT

**Figure 3:**
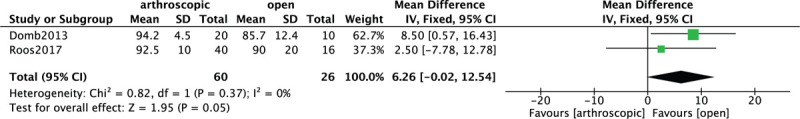
Forest plot of comparison: the Non-Arthritic Hip Score (NAHS) between arthroscopic treatment (AT) and open treatment (OT).

Comparison of postoperative NAHS between AT and OT was conducted between the 2 included studies,^[14,16]^ which enrolled 86 patients (60 patients receiving AT and 26 patients receiving OT), as shown in Figure 3. Heterogeneity testing showed that there was no heterogeneity between the studies (*P* = .37, *I*^2^ = 0%), so the fixed-effect model was used to pool the data for the 2 groups. The overall estimate showed that the difference was not statistically significant between the AT group and the OT group (MD = 6.26,95% CI = -0.02 to 12.54,*P* = .05).

### 3.6 Comparison of satisfaction rate between AT and OT

**Figure 4:**
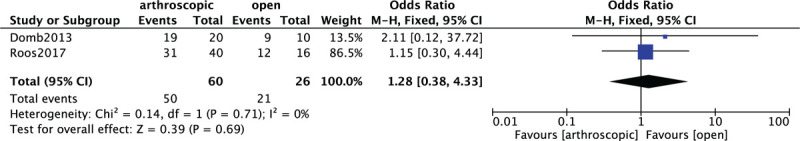
Forest plot of comparison: satisfaction rate between arthroscopic treatment (AT) and open treatment (OT).

Comparison of postoperative satisfaction rate between AT and OT was conducted among 2 included studies^[14,16]^ which contain 86 patients in Figure 4. A heterogeneity test showed that there was no heterogeneity among studies (*P* = .71, *I*^2^ = 0%), so the fixed-effect model was used. The overall estimate showed that the difference between the 2 groups was not statistically significant (OR = 1.28, 95% CI = 0.38–4.33, *P* = .69).

### 3.7 Comparison of VAS score between AT and OT

**Figure 5:**
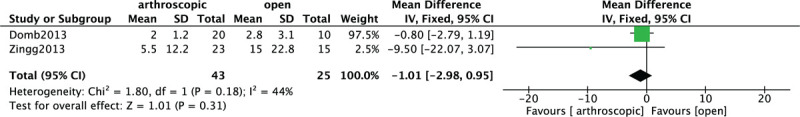
Forest plot of comparison: the Visual Analogue Scale (VAS) score between arthroscopic treatment (AT) and open treatment (OT).

In Figure 5, 2 included studies^[16,17]^ consisting of 68 patients (43 patients received AT and 25 patients received OT) investigated postoperative VAS score. Low heterogeneity among studies (*P* = .18, *I*^2^ = 44%) was found, so we used the fixed-effect model to pool the data. The overall estimate indicated that the pooled MD was -1.01 (95% CI = -2.98 to 0.95, *P* = .31), suggesting that AT and OT had no statistically significant difference.

### 3.8 Comparison of alpha angle and internal rotation angle between AT and OT

**Figure 6:**
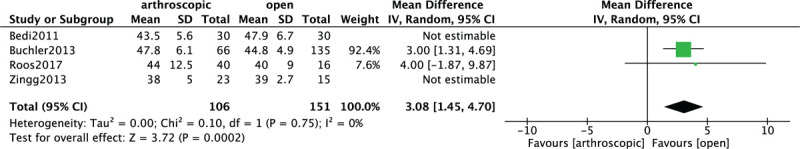
Forest plot of comparison: postoperative alpha angle between arthroscopic treatment (AT) and open treatment (OT).

**Figure 7:**
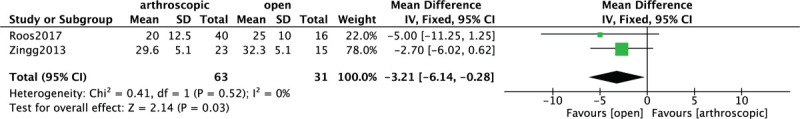
Forest plot of comparison: postoperative internal rotation angle between arthroscopic treatment (AT) and open treatment (OT).

Four included studies^[14,17–19]^ including 106 AT group cases and 151 OT group cases provided the data in terms of postoperative alpha angle. A heterogeneity test revealed that no heterogeneity existed among the studies (*P* = .75, *I*^2^ = 0%) and the fixed-effect model was used. A pooled analysis revealed that there was significant difference between AT and OT group (MD = 3.08, 95% CI = 1.45–4.70, *P* = .44) and OT group achieved better results (Fig. 6). Comparison of postoperative internal rotation angle between the 2 groups was conducted among 2 included studies,^[13,17]^ which contain 94 patients (63 patients received AT and 31 patients received OT) in Figure 7. No heterogeneity was found among studies (*P* = .52, *I*^2^ = 0%), so the fixed-effect model was used. The pooled result showed that the difference was statistically significant which favored OT group (MD = -3.21, 95% CI = -6.14 to -0.28, *P* = .03).

### 3.9 Comparison of complications between AT and OT

**Figure 8:**
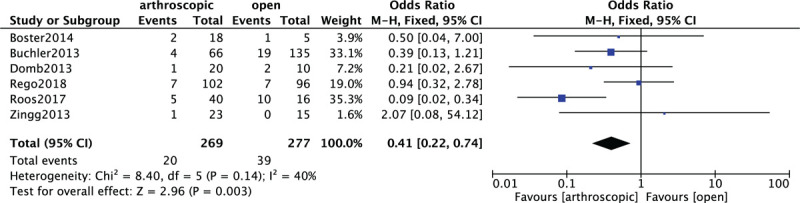
Forest plot of comparison: complications between arthroscopic treatment (AT) and open treatment (OT).

In Figure 8, 6 included studies^[13–18]^ consisting of 546 FAI patients (269 patients received AT and 277 patients received OT) reported complications. A low heterogeneity among studies (*P* = .14, *I*^2^ = 40%) was found, so we used the fixed-effect model. The overall estimate indicated that the pooled OR was 0.41 (95% CI = 0.22–0.74, *P* = .003), suggesting that the difference was statistically significant, and the complications of OT were higher than that of AT.

### 3.10 Sensitivity analysis and publication bias

**Figure 9:**
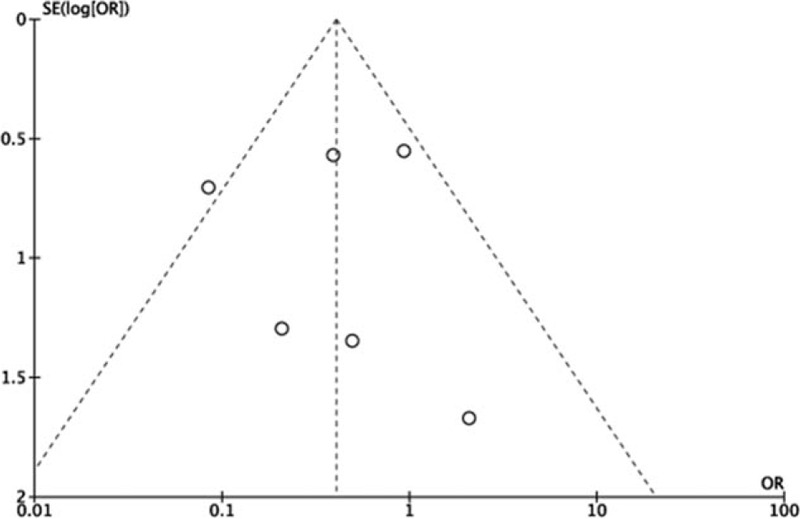
Funnel plot to test for publication bias. Each point represents a separate study for the indicated association. The vertical line represents the mean effects size. OR = odds ratio, SE = standard error.

We performed a sensitivity analysis to assess the stability of the pooled results. Among the most studies, the heterogeneity results were not obviously altered after sequentially omitting each study, indicating that our results were statistically reliable. Only for the pooled results of alpha angle, adding Bedi and Zingg's studies^[17,19]^ would increase the heterogeneity (*I*^2^ = 0% change into *I*^2^ = 85%), so we abandoned these 2 studies. The funnel plot of the included studies is shown in Figure 9. The points in the funnel plot were almost symmetrically distributed, indicating that the publication bias was not apparent.

## 4 Discussion

### 4.1 Summary of main results

In this study, we identified 7 observational studies for investigating the clinical outcomes and complications of AT versus OT. Our meta-analysis results showed that the differences were not statistically significant between the 2 interventions for mHHS, NAHS, VAS score, and satisfaction rate. The satisfaction rate was calculated according to good/excellent results patients evaluated. Good/excellent results were based on an mHHS greater than 80 points. However, a different result was discovered by alpha angle and internal rotation angle analysis. The difference of alpha angle and internal rotation angle was statistically significant between AT and OT, and the open surgery technique proved it had a higher efficacy.

Cam-type FAI was defined as alpha angle greater than 55° in Dunn 45° radiographs.^[20]^ Both of AT and OT reported mean postoperative alpha angle decreased to less than 50°, which were all recovered to normal angles. And overcorrection is unwelcomed since irreversible and may be an issue in terms of joint sealing.^[21]^ In the population in study by Zingg et al,^[17]^ a benefit of approximatively 18° from preoperatively to the 3 months and 1-year follow-up was seen. This benefit of internal rotation is in accordance with Kelly et al^[22]^ reporting an increase from 9.9° to 27.6° postoperatively at 3 months after arthroscopic decompression. However, according to other investigations, the gain of internal rotation may be less important or even without significant improvement.^[9,23]^ Although in these 2 open hip dislocation investigations was performed, we have not seen a significant difference either in absolute postoperative internal rotation or in the gain between the 2 techniques. A possible explanation for the varying results reported in the literature may be the potential positive influence of limited overcorrection of the deformities or compromising coexisting deformities such as reduced femoral torsion and centercollum-diaphyseal angle, and a deep acetabulum. An obvious advantage of arthroscopy over surgical dislocation is the reduced trauma to the trochanter and the soft tissues may shorten recovery after surgery. The trochanteric osteotomy requires healing time and restrictions for rehabilitation that may delay recovery.^[24]^ In study by Zingg et al,^[17]^ postoperative recovery in terms of hospital stay and time to return to work after AT is superior compared with OT, which proved AT's advantages.

The complications in 6 included studies should also be discussed. On the whole, 20 (7.4%) complications under AT was reported and 39 (14.1%) complications under OT was reported in 6 included studies,^[13–18]^ which showed that AT has the lower complications than OT. In study by Rego et al,^[13]^ 11 types of complications were identified in the AT and OT group: 2 adhesive capsulitis cases; delayed consolidation, pseudarthrosis, deep venous thrombosis and superficial wound infection which were only reported after OT; reversible pudendal nerve paresis, perineal cutaneous necrosis, compartment syndrome, hematoma, and heterotopic ossification, which were only reported after AT. For the study by Roos et al,^[14]^ 1 case (2.43%) presented deep venous thrombosis, 1 case (2.43%) presented heterotopic ossification, and 1 case (2.43%) presented transient paresthesia of the pudendal nerve. There were 2 cases (4.87%) with persistent pain. In the study by Boster et al,^[15]^ 1 patient in the arthroscopic group developed a superficial infection that was resolved with oral antibiotics. One patient in the open group reported persistent trochanteric pain. In the study by Büchler's,^[18]^ 4 patients (6.1%) of AT group underwent arthroscopic revision of intra-articular adhesions. In open group, 16 patients (12%) needed arthroscopic adhesiolysis and 3 patients (2.2%) underwent refixation of the greater trochanter for nonunion. In the study by Domb et al,^[16]^ hardware removal after OT and new-onset symptomatic internal snapping after AT were reported. In addition, 1 transient neuropraxia lateral femoral cutaneous nerve case after AT was noted in study by Zingg et al.^[17]^

### 4.2 Comparison with previous studies

To our knowledge, this is an updated systematic review and meta-analysis to compare the efficacy and complications of AT versus OT for FAI. A meta-analysis of 5 retrospective studies published in 2016 by Zhang et al^[11]^ found that hip arthroscopy resulted in higher NAHS and lower reoperation rates, but had less improvement in alpha angle in patients with cam osteoplasty, than open surgical dislocation. However, our study including more latest observational studies showed that AT had comparable effect in NAHS compared with OT. Zhang et al^[11]^ demonstrated no significant differences in complications by including only 2 articles, yet our study found that AT had lower complications than OT by pooling 6 articles. In addition, as a supplement, our study also compared satisfaction rate, VAS score, and postoperative internal rotation angle between AT and OT, which enriched our results compared with previous meta-analysis.

### 4.3 Limitations of the study

Some limitations of this study should be noted. First, the small sample size might have affected the significant difference between the 2 surgical procedures. Second, our study ignored the diversity of used diagnostic criteria and etiology of the disease, and further research is needed to discover whether these conclusions apply to patients with varying degrees of FAI. Last but not least, the included studies were all observational studies and not RCTs, and they largely relied on retrospectively collected data, resulting in a relatively high risk of selection bias. More large-sample, multicenter, high-quality, RCTs are needed to verify the outcomes of this meta-analysis.

## 5 Conclusion

Both AT and OT had benefits in FAI. AT had comparable effect in mHHS, NAHS, VAS, and satisfaction rate as well as lower complications than OT. For postoperative alpha angle and internal rotation angle, OT achieved better results. In view of the heterogeneity and different follow-up time, whether these conclusions are applicable should be further determined in future studies.

## Author contributions

YMR, QHY, and MQT conceived the design of the study. QHY and YMR performed and collected the data and contributed to the design of the study. QHY and YMR analyzed the data. YMR, YHZ, and MQT prepared and revised the manuscript. All authors read and approved the final content of the manuscript.
